# Disseminated Phaeohyphomycosis Due to *Alternaria alternata* in a Liver Transplant Recipient: Successful Treatment With Isavuconazole, a Case Report and Literature Review

**DOI:** 10.1155/crit/9985652

**Published:** 2026-07-16

**Authors:** Steven Tapia-Villacis, Jimena Prieto, Martín López, Julio Medina

**Affiliations:** ^1^ Unidad Académica de Enfermedades Infecciosas, Facultad de Medicina, Universidad de la República, Montevideo, Uruguay, universidad.edu.uy; ^2^ Programa Nacional de Trasplante Hepático (Unidad Bi Institucional, Hospital Central de Las Fuerzas Armadas, Hospital de Clínicas Dr. Manuel Quintela), Montevideo, Uruguay

**Keywords:** *Alternaria*, dematiaceous, isavuconazole, phaeohyphomycosis, transplantation

## Abstract

Phaeohyphomycoses are a heterogeneous group of diseases caused by several genera of pigmented fungi, also called dematiaceous fungi. These organisms are increasingly recognized as emerging opportunistic pathogens in immunocompromised patients, particularly solid organ transplant recipients. Among them, *Alternaria* is the most frequently associated genus, with clinical manifestations ranging from superficial to disseminated infections. Treatment options are limited and often complicated by toxicity and drug interactions, particularly in the posttransplant setting. We report what is, to our knowledge, the first case of disseminated alternariosis in a liver transplant recipient in Uruguay, successfully treated with isavuconazole. This case highlights the importance of early recognition and tailored antifungal therapy in transplant patients presenting with unusual cutaneous and pulmonary manifestations.

## 1. Introduction

Phaeohyphomycosis refers to infections caused by dematiaceous (melanin‐producing) fungi, widely distributed in the environment as saprophytes in soil, air, and plants. These fungi have emerged as important opportunistic pathogens in immunocompromised hosts, especially in solid organ transplant (SOT) recipients [1, 2].

*Alternaria* spp.—particularly *Alternaria alternata*, *Alternaria infectoria*, and *Alternaria tenuissima*—are among the most implicated genera [1, 3]. Clinical presentations vary from superficial cutaneous infections to invasive and disseminated disease [4–11]. Management includes antifungal agents, such as amphotericin B, triazoles, and echinocandins, occasionally combined with surgical interventions [3–8].

We present the case of a liver transplant recipient with *A. alternata* infection, which resolved with antifungal therapy including isavuconazole—a triazole rarely reported in the treatment of phaeohyphomycosis.

## 2. Clinical Case

A 38‐year‐old woman from rural Salto, Uruguay, underwent orthotopic liver transplantation in 2001 for cirrhosis secondary to autoimmune hepatitis. In 2015, she experienced disease recurrence, requiring escalation of immunosuppressive therapy to include tacrolimus, prednisone, mycophenolate mofetil, and everolimus.

In October 2021, due to persistent allograft dysfunction despite intensified immunosuppression, she was admitted to the Interdisciplinary Liver Transplant Unit at the Army Forces Hospital in Montevideo for evaluation of possible rejection. Given the refractory nature of the rejection, anti‐CD20 monoclonal antibody (rituximab) was considered as salvage therapy.

During admission, physical examination revealed multiple indurated, nonconfluent nodules with superficial desquamation and occasional serous discharge involving the pretibial region of the right lower limb, extending toward the knee and distal thigh (Figure 1). The patient reported a progressive evolution of these lesions over more than 12 months. She recalled minor trauma at the site of the initial lesion due to a peck from a domestic chicken, as well as frequent contact with weeds in her home garden, where she cultivated tomatoes.

**Figure 1:**
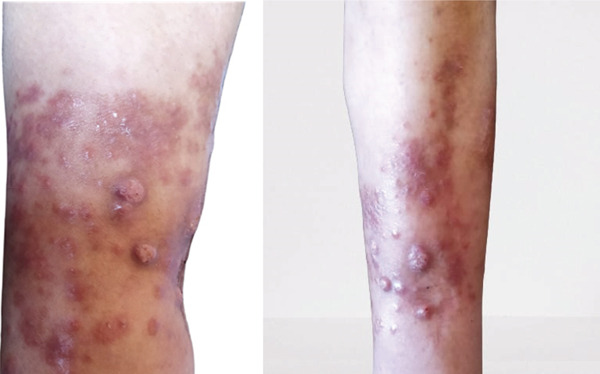
Cutaneous lesions on the right lower limb at initial evaluation. Multiple erythematous to violaceous papules and nodules were observed, some coalescing into plaques, with smooth or verrucous surfaces and no signs of ulceration. Lesions involved the pretibial region, extending toward the knee and distal thigh. Courtesy: Prieto J MD. MsC.

Surgical excision of the lesions was performed. Histopathology showed a chronic inflammatory process with dermal microabscesses, and Gomori–Grocott staining revealed filamentous fungal structures. Culture grew a dematiaceous mold, identified as *A. alternata* by matrix‐assisted laser desorption/ionization time‐of‐flight mass spectrometry (MALDI‐TOF MS). No molecular sequencing was performed.

Given the potential risk of dissemination in an immunocompromised host, a comprehensive imaging assessment was performed. Computed tomography revealed radiological abnormalities involving the bilateral paranasal sinuses, as well as multiple bilateral pulmonary infiltrates characterized by ground‐glass opacities and ill‐defined centrilobular nodules, predominantly affecting the lower lobes, with peribronchovascular and subpleural distribution (Figure 2A–D). No cavitary lesions or pleural effusion were observed. The sinus findings were interpreted as probably associated with the same infectious process in the overall clinical context. However, otorhinolaryngological evaluation was not performed, and no direct microbiological sampling of the paranasal sinuses was obtained.

**Figure 2:**
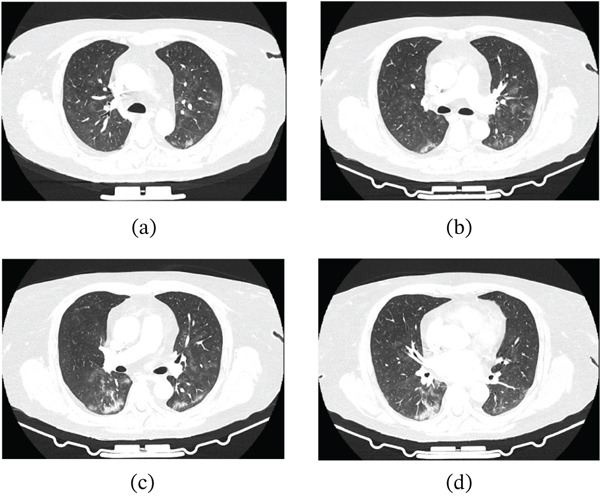
(a–d) Axial chest computed tomography (CT) scans in lung window showing multiple bilateral ground‐glass opacities with ill‐defined centrilobular nodules, predominantly affecting the lower lobes. A peribronchovascular and subpleural distribution was observed, with no evidence of cavitation or pleural effusion. Images courtesy of Dr. Javier Lagreca.

Fiberoptic bronchoscopy with bronchoalveolar lavage (BAL) was performed. BAL fungal culture was negative. A multiplex molecular panel for lower respiratory tract pathogens (BIOFIRE FilmArray Pneumonia Panel, bioMérieux) was also negative, and BAL galactomannan antigen testing was negative. No fungal‐specific molecular testing for dematiaceous fungi was performed on the BAL sample, as these tests are not currently available in our country.

Although pulmonary involvement was not microbiologically proven, it was considered probable based on the histopathologically and microbiologically confirmed cutaneous *A. alternata* infection, the presence of bilateral pulmonary infiltrates with a pattern compatible with fungal infection in an immunocompromised host and the exclusion of common respiratory pathogens. Based on these findings, antifungal therapy was initiated.

Initial antifungal therapy consisted of intravenous voriconazole (6 mg/kg loading dose, followed by 4 mg/kg every 8 h) combined with caspofungin (70 mg loading dose, followed by 50 mg/day). During treatment, close monitoring and adjustment of the immunosuppressive regimen were required. Calcineurin and mTOR inhibitors were tapered to minimize drug–drug interactions and potential toxicity, particularly in relation to voriconazole. On Day 7 of treatment, voriconazole was discontinued because of worsening liver function and suspected drug‐induced hepatotoxicity.

Caspofungin was continued, and oral terbinafine (100 mg every 12 h) was added for the following 14 days. Thus, the patient received caspofungin for a total of 21 days, initially in combination with voriconazole for 7 days, and subsequently in combination with terbinafine for 14 days. Compassionate‐use authorization was then obtained for isavuconazole, as it was not registered in the national formulary. Isavuconazole was selected because of its more favorable safety and pharmacokinetic profile, particularly in the setting of impaired liver function and concomitant use of multiple immunosuppressive agents. Antifungal therapy was continued with oral isavuconazole (200 mg every 8 h for 48 h for loading dose, followed by 200 mg every 24 h) for a total duration of 6 months.

The patient showed significant clinical improvement, with complete regression of cutaneous lesions and radiological resolution of pulmonary infiltrates after 8 weeks of isavuconazole therapy (Figure 3). No evidence of hepatotoxicity was documented, and treatment was well tolerated. After completing the 6‐month course of antifungal therapy, she remained under clinical follow‐up for 12 months, with no evidence of recurrence. No follow‐up microbiological cultures were obtained, as there were no active cutaneous lesions or clinical features suggesting persistent or recurrent infection, and follow‐up computed tomography showed radiological improvement. The patient remains asymptomatic and continues to be followed at the transplant infectious diseases clinic.

**Figure 3:**
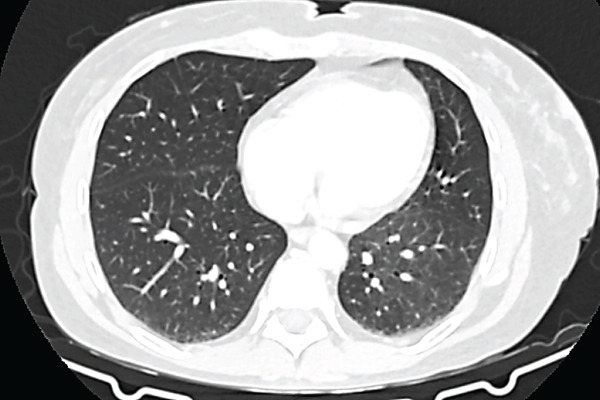
Follow‐up chest computed tomography (CT) scan in lung window performed after antifungal therapy. A marked radiological improvement was observed, with resolution of the previously described ground‐glass opacities and nodular infiltrates. No cavitary lesions or pleural effusion were identified. These findings were consistent with a favorable response to antifungal therapy.

## 3. Discussion

*Alternaria* spp. have emerged as opportunistic pathogens, particularly in immunocompromised individuals [3, 5, 12, 13]. Common predisposing factors include contact with soil, trauma, corticosteroid therapy, and diabetes [13]. In addition, immunosuppression and Cushing′s syndrome are recognized contributors to increased susceptibility [2, 10]. Recent studies also highlight the role of innate immune defects, such as CARD9 mutations and Th17 pathway deficiencies, in the pathogenesis of these infections [14].

In the setting of solid organ transplantation, a recent systematic review of 180 case reports showed a predominance of male patients (72%), with a median age of 54 years. Kidney transplant recipients represented 61% of cases, with a median onset of infection at 31 months posttransplantation [5].

In SOT recipients, cutaneous manifestations are the most frequently reported, although sinusitis, pneumonia, and central nervous system involvement have also been described [7–9]. The TRANSNET study identified *Alternaria* as the leading cause of phaeohyphomycosis in transplant recipients, with over half of the cases presenting with disseminated disease [4]. Vennewald and Molina proposed three clinical forms of phaeohyphomycosis based on the presumed mechanism of acquisition: (a) exogenous superficial, secondary to preexisting dermatopathy; (b) exogenous unilocular, typically posttraumatic; and (c) exogenous multilocular or disseminated, often resulting from hematogenous spread [11].

The present case is notable for its chronic cutaneous progression, combined with radiological paranasal sinus abnormalities and probable pulmonary involvement, in the context of complex immunosuppression. Although the paranasal sinus abnormalities could not be microbiologically attributed to *Alternaria* spp., as otorhinolaryngological evaluation and direct sinus sampling were not performed, sinus involvement has been described as part of the clinical spectrum of *Alternaria* infection [7–9]. Similarly, pulmonary involvement was not microbiologically proven; however, the presence of bilateral pulmonary infiltrates with a pattern compatible with fungal infection in an immunocompromised host, the exclusion of common respiratory pathogens, and the radiological resolution after antifungal therapy supported its interpretation as probable pulmonary involvement. The patient received four immunosuppressive agents, including calcineurin and mTOR inhibitors, and was under evaluation for anti‐CD20 monoclonal antibody therapy at the time of diagnosis. This profound immunosuppressive status likely contributed to the disseminated presentation observed.

Direct microscopy and histopathology, in conjunction with fungal culture, remain essential diagnostic methods. Direct examination allows visualization of darkly pigmented hyphae invading tissue, serving as the diagnostic cornerstone. Culture is necessary for species‐level identification, although its performance is variable [2, 3, 13]. Histological analysis may reveal spared epidermis, dermal granulomas with or without central abscesses, and a mixed inflammatory infiltrate composed of macrophages, plasma cells, and neutrophils [11]. In recent years, MALDI‐TOF MS has been introduced as a rapid diagnostic tool for species identification based on specific protein profiles [15]. Although highly effective, it requires viable, actively growing cultures with a sufficient biomass for accurate identification.

Therapeutic decisions depend on the clinical presentation, anatomical location, number of lesions, and the presence or absence of dissemination [3–7]. Although there are no standardized treatment guidelines due to the rarity and heterogeneity of phaeohyphomycosis, a recent meta‐analysis in SOT recipients suggests that the most used agents include polyenes (amphotericin B), triazoles (excluding fluconazole), and echinocandins, with a median treatment duration of 5 months [5]. The choice of antifungal regimen is often influenced by the severity and extent of disease, drug toxicity profiles, and potential pharmacological interactions. Allylamines such as terbinafine have also been reported as part of combination therapy or monotherapy in patients on immunosuppressive regimens, owing to their favorable interaction profile with calcineurin inhibitors [9].

Isavuconazole, like other triazoles, inhibits 14‐alpha‐lanosterol demethylase (CYP51), an enzyme essential for ergosterol synthesis in fungal cell membranes. It has broad‐spectrum activity, including efficacy against certain filamentous fungi resistant to other triazoles [16]. Isavuconazole is currently approved for treating invasive infections caused by *Aspergillus* spp. and *Mucorales* [17, 18]. A subanalysis of the VITAL Phase III study evaluating isavuconazole for rare fungal infections—including dematiaceous fungi—demonstrated favorable safety and efficacy, though clinical experience remains limited [16].

In the present case, isavuconazole was selected due to its broad antifungal spectrum, more predictable pharmacokinetics, reduced hepatotoxicity, and lower risk of interactions with immunosuppressive agents, particularly in the context of multiple immunosuppressive therapies and impaired liver function. The excellent clinical and radiological response observed with the sequential use of echinocandin–terbinafine followed by isavuconazole—despite the limited data supporting this approach in phaeohyphomycosis [19, 20]—is particularly noteworthy.

The prognosis of phaeohyphomycosis varies with disease severity. Although cases limited to the skin generally have favorable outcomes, disseminated forms are associated with significantly higher mortality rates—reaching up to 70% in immunocompromised hosts [4]. Surgical debridement may be required in certain cases, particularly those with localized cutaneous lesions [1, 2, 5, 6].

Early diagnosis and individualized antifungal therapy are critical for managing these rare fungal infections, particularly in immunosuppressed patients. This case highlights the importance of recognizing dematiaceous fungi as potential pathogens in individuals under intense immunosuppression and epidemiological exposure and supports the consideration of isavuconazole as a therapeutic alternative in settings with limited treatment options. To our knowledge, this represents the first documented use of isavuconazole for phaeohyphomycosis in a transplant recipient in Uruguay.

## Funding

No funding was received for this manuscript.

## Conflicts of Interest

The authors declare no conflicts of interest.

## Data Availability

The data that support the findings of this study are available on request from the corresponding author. The data are not publicly available due to privacy or ethical restrictions.
